# Clinician-centric diagnosis of rare genetic diseases: performance of a gene pertinence metric in decision support for clinicians

**DOI:** 10.1186/s13023-020-01461-1

**Published:** 2020-07-22

**Authors:** Michael M. Segal, Renee George, Peter Waltman, Ayman W. El-Hattab, Kiely N. James, Valentina Stanley, Joseph Gleeson

**Affiliations:** 1 SimulConsult Inc, Chestnut Hill, MA USA; 2 Department of Neurosciences, University of California San Diego, La Jolla, CA USA; 3 Rady Children’s Institute for Genomic Medicine, Rady Children’s Hospital, San Diego, CA USA; 4 Rockefeller University, New York, NY USA; 5 current address Department of Systems Biology, Columbia University, New York, NY USA; 6 Department of Clinical Sciences, College of Medicine, University of Sharjah, Sharjah, United Arab Emirates

**Keywords:** Rare disease diagnosis, Diagnostic decision support system, Artificial intelligence, Genomic analysis, Copy number variation

## Abstract

**Background:**

In diagnosis of rare genetic diseases we face a decision as to the degree to which the sequencing lab offers one or more diagnoses based on clinical input provided by the clinician, or the clinician reaches a diagnosis based on the complete set of variants provided by the lab. We tested a software approach to assist the clinician in making the diagnosis based on clinical findings and an annotated genomic variant table, using cases already solved using less automated processes.

**Results:**

For the 81 cases studied (involving 216 individuals), 70 had genetic abnormalities with phenotypes previously described in the literature, and 11 were not described in the literature at the time of analysis (“discovery genes”). These included cases beyond a trio, including ones with different variants in the same gene. In 100% of cases the abnormality was recognized. Of the 70, the abnormality was ranked #1 in 94% of cases, with an average rank 1.1 for all cases. Large CNVs could be analyzed in an integrated analysis, performed in 24 of the cases. The process is rapid enough to allow for periodic reanalysis of unsolved cases.

**Conclusions:**

A clinician-friendly environment for clinical correlation can be provided to clinicians who are best positioned to have the clinical information needed for this interpretation.

## Background

The number of rare diseases described in the literature has increased dramatically in recent decades, primarily due to advances in our understanding of genetics. We are at a crossroads in deciding how to use this information.

One approach is to submit DNA to labs to use massively parallel sequencing to identify genetic diagnoses, but this approach is difficult to implement in an optimal way because clinicians provide limited information to the lab, which is therefore not well equipped to do the clinical correlation. Furthermore, because the clinical information is typically submitted before the genomic sequencing, there is little opportunity for the lab to bring into the clinical correlation information prompted by the unusual gene variants found in sequencing.

Another approach is for clinicians to remain at the center of clinical correlation, drawing on the detailed clinical characterizations in the primary literature as well as comprehensive reviews in the literature. However, such clinical correlation is difficult for clinicians to perform because of the complexity involved in dealing with thousands of variants and thousands of diseases.

We suggested solving this problem by empowering the clinicians who are most familiar with the patient to take a central role in the clinical correlation step [1]. We implemented such capabilities using software that clinicians already use for clinical diagnostic decision support, augmented by analysis of variants and known associations between genes and diseases, linking back to clinical resources to assist the clinician in assessing diagnostic possibilities. We ran an initial test using 20 cases with pathogenic single nucleotide variants (SNVs) in genomic trios [2]. An early version of the system was also tested in the CLARITY genome-analysis competition and was the most clinician-centric of the analyses and it performed by far the fastest [3].

We now test this model more widely and systematically, and test extension of this model to include large copy number variants (CNVs). CNVs are typically assessed in a separate analysis using microarray technology performed before doing genomic sequencing [4]. We examine here whether we could use CNVs and SNVs, both derived from genomic sequencing, in a single test, which could offer a process for clinical correlation that is more efficient.

We also test the utility of extending such SNV and CNV analysis beyond the trio, thereby reducing the number of plausible genetic abnormalities.

## Methods

For “beyond-the-trio” cases, all cases from the Gleeson cohort of ~ 10,000 families were selected if there had been sequencing beyond the trio and a causative gene had met ACMG guidelines for reporting back to the family [5]. The Gleeson group focuses on neurological disorders, and all cases analyzed had at least one neurological finding. The diagnosis had been determined by the Gleeson group in a manual process typically requiring many hours, extensive expertise and wider sequencing within the family to establish correlation. In all cases the putative causal variant was confirmed by Sanger sequencing. Similarly, all cases with large CNVs were selected.

Most cases were ones in which the gene-disease relationship had already been described in the literature, but some had no such description, allowing analysis of situations in which clinical correlation did not yield an answer.

### SNV information in variant tables

Variant data for the individuals in a case were combined in annotated variant tables in the format described [6].

Exome sequencing reads were mapped to the hg38 version of the human reference genome using bwa-mem with default parameters [7]. Duplicates were marked with Picard’s MarkDuplicates v1.128 [8] and indels were realigned using GATK’s IndelRealigner v3.5 [9]. Variant calling for SNVs and indels was performed according to GATK’s best practices by first calling variants in each individual sample and then genotyping them jointly across all individuals used in this study.

Variants were annotated with the Variant Effect Predictor [10] to include:

- Functional effect (e.g. synonymous, stop gain, etc.)
- Allele frequencies from the 1000 Genomes Project. the Genome Aggregation Database (gnomAD), and the Greater Middle East variome [11]
- Pathogenicity predictions using SIFT, PolyPhen, and MutationTaster
- Conservation assessments using GERP, PhyloP, and PhastCons.
- Variome “share scores” of the number of times a variant was observed in the homozygous and heterozygous state in the Gleeson Lab cohort.

Variants were filtered to include those that were predicted to affect protein function (frameshift, non-synonymous, stop gain, splice site, CNV) and rare (< 0.1% allele frequency in gnomAD and < 1% allele frequency in the Greater Middle East variome [11].

### CNV information in variant tables

CNV calls were generated with XHMM according to the protocol of Fromer and Purcell [12]. The CNVs were then annotated with overlapping genes and their frequency from the Exome Aggregation Consortium (ExAC) database [13].

CNV data was added to the same variant tables as SNVs by the following changes to the previous format for the variant tables:

- Instead of listing one HGNC gene symbol for a variant, all genes in the interval were listed (e.g., RN7SL853P,GOLGA6B)
- Instead of listing a single chromosomal position, an interval was specified (e.g., 15:72954547–72,958,739)
- Instead of listing functional effects such as frameshift, CNV abnormalities were described as DEL or DUP.

### Pedigree files

Pedigree files in standard format [14] specified information about parents, sex, and affected status. For a trio, relevant information was already in the patient clinical data file.

### Genome-Phenome analyzer software

The cases were analyzed using the SimulConsult Genome-Phenome Analyzer [15], a diagnostic decision support program that helps clinicians assess the diagnostic possibilities for a patient. The core of the tool is the ability to assess clinical findings, but it also includes the ability to analyze a genomic variant table and deduce which genetic variants can contribute to genetic disease. By combining what is known about the patient together with the known genotypes of diseases (the “genome”) and the known phenotypes of diseases (the “phenome”), it assists with the “genome-phenome analysis” needed for genomic diagnosis. For this study, the patient data was loaded into the software in the form of the following files prepared by the Gleeson group: the patient findings file, the annotated variant file, and for cases beyond the trio, a pedigree file.

The design and function of the genome-phenome analysis software and the evaluation of its analysis for SNVs in trios have been described previously [2, 16–19]. Briefly, the software compares a patient’s findings to known diseases in the software’s human curated database that includes information from many resources such as textbooks in various fields, review articles such as all relevant reviews in Orphanet Journal of Rare Diseases, and many original articles. The software provides a differential diagnosis and suggests further findings useful in making a diagnosis [16], both before and after the genome information is available. Findings can be present (with onset at a particular age or by a particular age) or absent, allowing the appropriate pattern matching with information about findings in diseases in the database, including the onset and disappearance ages for each finding in each disease [18]. The disease descriptions in the database include mode of inheritance, but the software considers inheritance in a hypothesis-independent way based on family history for the patient, including consanguinity and affected status of other family members [17].

### Identifying abnormal gene zygosities

The variant table is analyzed using the software as described previously [2]. Briefly, variant severity scores (0–5) were assigned based on the annotations. These variant-level scores were combined to assign severity scores at the level of gene zygosity (i.e., biallelic versus monoallelic, treated separately because of their different clinical associations with disease), using the information about which individuals were affected and which had the variants [2, 19].

The ability to process variant tables beyond the trio was achieved by dividing the case group (typically a family) into a set of trios or partial trios (e.g., a case with proband + sister + their parents + a cousin was divided into 3 trios: proband-mother-father, sister-mother-father, and cousin alone). The software then compared each trio at the gene zygosity level, looking for abnormal zygosities that fit the affected status of all individuals in each trio. This analysis at the level of the gene zygosity, not the gene variant, allows identifying an abnormal zygosity even if individuals in different trios had different abnormal variants in the same gene, as was seen in many cases of unrelated individuals.

Each abnormal gene zygosity is assigned a pertinence score according to how different the differential diagnosis would have been if the zygosity had not been abnormal, described in detail previously, including how the pertinence score is affected by the severity score assigned to each gene zygosity [2, 19].

The software was re-written from the previous version [2] to retain the original algorithms but have a client-side interface using the Angular JavaScript / TypeScript framework and the core analysis is performed on a server. The HIPAA-compatible server uses a RESTful approach in which it retains no information about the patient between clicks by the user. Computational times were < 1 s to process the case and < 10 s to upload the full variant table to the server (done once per case).

### CNV analysis

CNV abnormalities were analyzed in 2 ways. One was by considering each gene in the interval to have a variant of maximal (5) severity. The other was by assessing the CNV for overlap with the 185 chromosomal disorders in the database (e.g., 15q13.3 microdeletion) and assigning a severity score (1–5) according to the degree of base overlap.

### Patient findings file

Patient finding files, consisting of lists of findings in the individual designated as the index case, were prepared by the Gleeson group before the genomic analysis. Each finding was linked to one or more Human Phenotype Ontology (HPO) codes and one or more Unified Medical Language System (UMLS) codes. As in previous work, both clinical and lab findings and both pertinent positive and pertinent negative findings were used, and onset ages for findings were included when available [2].

The database was increased substantially since the previous study [2]: coverage of diseases was increased by 45% (4912 to 7111) and the number of genes was increased by 43% (2734 to 3903). This coverage included all genes with germline changes convincingly associated with human disease at the time of analysis; (the database typically only includes somatic changes if they arise early in development).

## Results

A total of 81 cases were selected, representing exomes from 216 individuals (Table 1). 70 cases had abnormal gene zygosities with phenotypes previously described in the literature; the genes with pathogenic variants were (with numbers greater than one indicated in parentheses): AGTPBP1, AHI1, ALG1, ALS2, AMPD2 (2), ARG1, ASPM (3), ATP8A2, BBS12 (2), CA8, CC2D2A, CENPJ, CEP120, CLN6 (2), CPLANE1, CSPP1, ENTPD1, ERCC8, ERLIN1, ERLIN2, GAMT, GEMIN4, GJC2, GLDC, GRID2 (2), GRIK2, HEXB, HSPD1, KATNB1, KCTD7, KIF7, LNPK, MCPH1, MKS1, NDUFV1, NGLY1, NPC1, NPHP3, NPHP4, NT5C2, PAFAH1B1, PCDH12, PEX1, PGAP2, PGAP3, PIGC, POMT2, PYCR2, RNASEH2B, TBC1D20, TFG, THOC6, TMEM138, TOE1, TPP1, TRAPPC9, TSEN2, TSEN54, TTC19, VPS13B (2), WDR62, WWOX (2). In 11 cases the gene associations were not described in the literature at the time of analysis (“discovery genes”). In 24 of the cases, the genomic data included CNVs as well as SNVs.

**Table 1:** The 81 cases with 216 individuals used in the study

Type of case	Cases	Known gene	Discovery gene	Individuals per case		Rank of correct (known gene cases)		Number of zygosities (known gene cases)	
				Average	Range	Average	Range	Average	Range
SNV in nuclear family	18	15	3	2.39	2–4	1.13	1–3	12.60	1–21
SNV variant shared	19	19	0	3.11	2–6	1.16	1–4	2.63	1–10
SNV gene shared	20	15	5	3.60	2–6	1.00	1	1.27	1–2
CNV in nuclear families	24	21	3	1.75	1–3	1.14	1–3	87.00	1–790
TOTAL	81	70	11	2.67	1–6	1.11	1–4	29.79	1–790

**Fig. 1:**
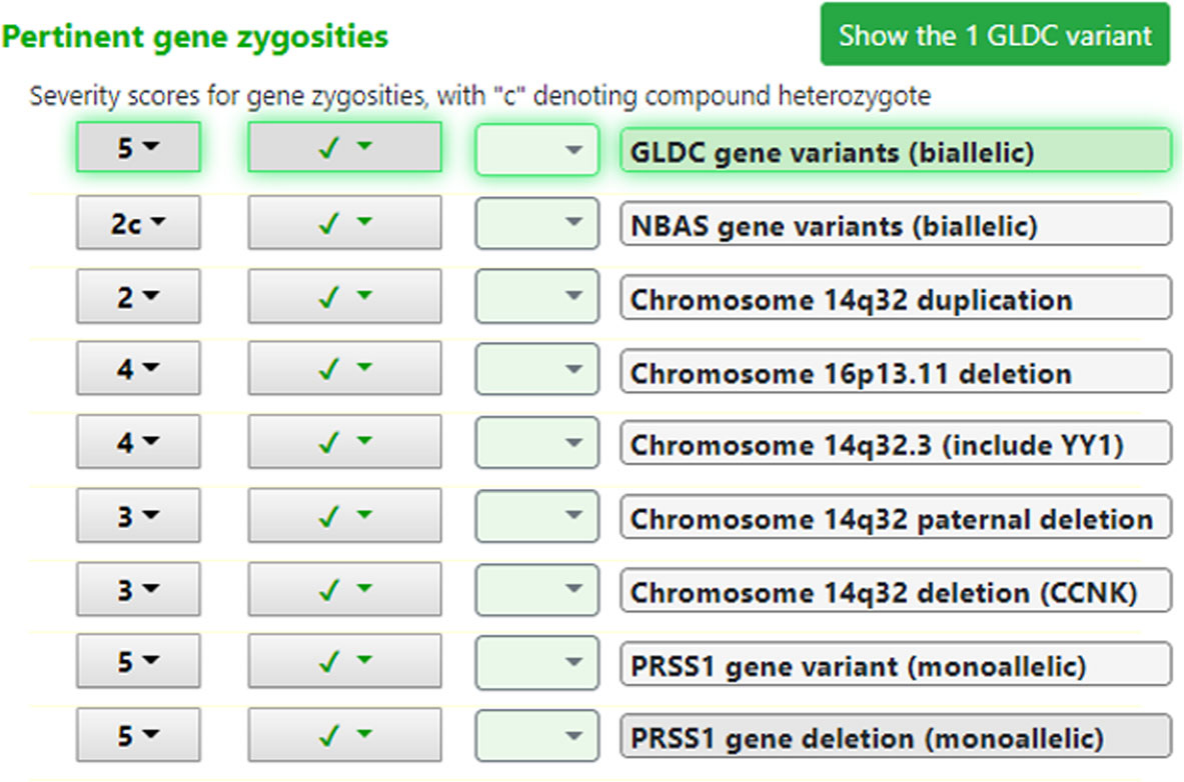
**Genome-Phenome Analysis display including both SNV and CNV results**. Display of gene zygosities that fit with the variants and affected status of all individuals used. Numbers to the left are severity scores for each zygosity (e.g., NBAS gene variants, biallelic, with “c” denoting compound heterozygote). Zygosities are *not* ranked by severity score; instead they are ranked by the pertinence metric, here 100% for biallelic *GLDC* gene variants (denoted by light green shading) and 0% for the other zygosities and chromosomal abnormalities shown that represent other possible genetic diagnoses but are much lower in pertinence. The pertinence metric depends on both the severity of the gene zygosity and the clinical findings entered for the proband. The GLDC and PRSS1 variants were derived from a deletion region and the NBAS variants were derived from SNPs. Clicking on the “Show the 1 GLDC variant” button shows a mini variant table with that one variant location and an explanation of how the severity score was determined for that variant (not shown here). The check marks denote variants that were found, using the convention used for all findings, where for example, @6 m for a clinical finding would denote that the clinician had entered that finding as having onset at 6 months of age (not shown here). The boxes between the check marks and the zygosities are used to denote the clinician’s choice of a gene zygosity to report as pathogenic (not used in this illustration)

There were 57 cases with SNVs only, divided into 3 groups depending on familial relationships: *nuclear family* (all were beyond the trio by virtue of having more than one sibling), *variant shared* (beyond nuclear families but with the same pathogenic variant), and *gene shared* (unrelated, with different variants in the same gene). Cases with CNVs were all within nuclear families, but 7 were beyond the trio by virtue of including a sibling. The number of zygosities (i.e., monoallelic versus biallelic) in the genome-phenome clinical correlation (e.g., Fig. 1) and rank of the gene zygosity that was correct (1 = top) are shown only for “known gene cases”; i.e., cases with a known gene-phenotype association in which a genome-phenome correlation can be done

**Fig. 2:**
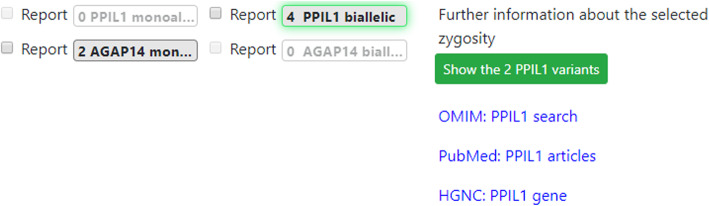
**Gene discovery display**. This display shows gene zygosities not associated with any published genetic condition. In the case shown here, only 2 discovery gene candidates were found, ranked by severity score (no pertinence metric is possible for gene zygosities with no known clinical phenotype). The *PPIL1* gene (biallelic) variants were reported as causative

The analysis had two output displays relevant to this study. One was a “genome-phenome analysis list” of gene zygosities with known genome-phenome associations, with severity scores (Fig. 1). The other was a list of “discovery genes”, which are gene zygosities that were abnormal but not associated with any published genetic condition (Fig. 2).

In the genome-phenome analysis display, the abnormal gene zygosities are ranked by their “pertinence” (Fig. 1, light green shading of the variant name of the display), not by severity. The average number of zygosities listed for consideration was 12.6 for SNVs in nuclear families, lower when less related individuals were included and higher when large CNVs were included (Table 1). Pertinence is computed by measuring how different the differential diagnosis would be without that zygosity finding being abnormal [2, 19]. Pertinence scores are normalized to the highest pertinence score for all findings, including clinical findings, with the highest possible pertinence score thereby 100% as seen in Fig. 1.

### Genome-Phenome analysis cases

For the 70 non-discovery cases, in 100% of cases the gene zygosity deemed causative by the Gleeson group was identified by the analysis. In 66 of these 70 cases it was ranked as #1 in the genome-phenome analysis list. In the 4 other cases the rankings were 2, 3, 3, and 4, with other abnormal gene zygosities ranked higher. Overall, the average ranking for the correct gene zygosity was 1.11 (Table 1). The smallest numbers of zygosities were seen in cases of unrelated individuals having non-shared variants in the same gene (rank of correct diagnosis 1.00; 1.27 zygosities in total; Table 1), cases that also had the highest number of individuals per case (3.60; Table 1).

In the 4 cases in which ranking was not #1, the average number of individuals per case was 1.75, versus 2.67 for all cases. The experimental design did not allow for additions to the patient findings after the information about the abnormal gene zygosities was available, as would be done in clinical practice. The experimental design also did not allow for additions of findings to the database for relevant diseases that had not been previously curated in the database. Such changes were simulated and resulted in large improvements in pertinence and are standard practice in clinical use of the Genome-Phenome Analyzer, but the results we report are without any such changes so as to eliminate the bias that could be introduced by such improvements.

### Identifying the correct gene zygosities

The results above demonstrate that even without adding further clinical data or database information, in the cases with a known gene, the #1 gene is ranked perfectly or near perfectly. But the challenges faced in genome analysis are complicated because there are situations in which the pathogenic gene is not in the genome-phenome display at all (Fig. 1), represented here by cases in which the answer is in the discovery gene display (Fig. 2). In the 11 gene discovery cases, there was often at least one gene zygosity in the genome-phenome analysis listing (average 9.1; range 0–43, with 6 of 11 having non-zero numbers of zygosities). In such cases the zygosity ranked #1 in the genome-phenome display had very low pertinence. However, despite the clinical implausibility of the zygosities in these genome-phenome analysis lists, these cases serve to illustrate the task faced by the person interpreting the genome: to assess whether the zygosities listed in the genome-phenome display were pathogenic (but having unusually low pertinence in the genome-phenome display) versus irrelevant (with the answer being among discovery genes or no answer in the exome at all).

To guide such assessments, we examined the #1 ranked pertinence scores for all 81 cases. We divided them into “positives” (the 66 cases in which the correct gene zygosity was ranked #1 in the genome analysis) and “negatives” (the 15 cases in which the correct gene zygosity was not ranked #1 in the genome-phenome analysis: i.e., 4 cases in which the gene zygosity was on the genome-phenome analysis list but not #1 plus 11 cases in which the pathogenic gene was a discovery gene and thus not on the genome-phenome analysis list at all).

**Fig. 3:**
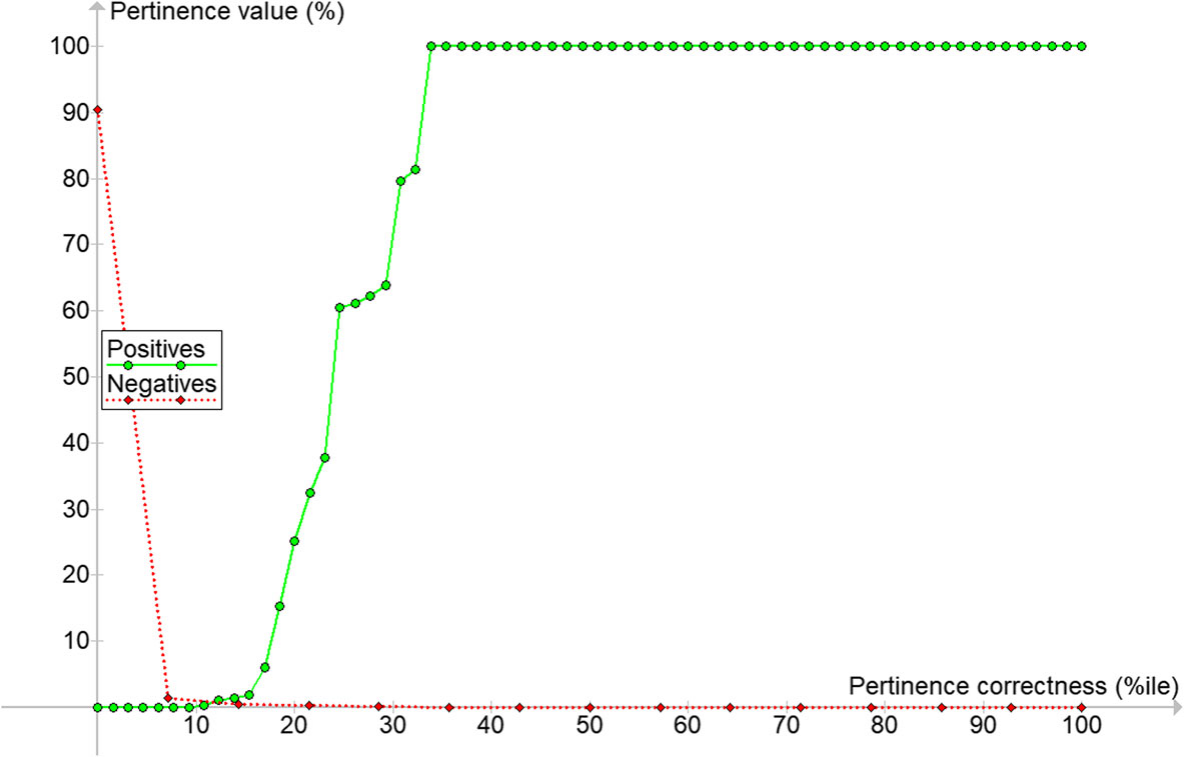
**Pertinence values for positives and negatives**. Positives are the 66 cases in which the correct (reported) gene was #1 in the genome-phenome analysis output (e.g., Fig. 1); in 67% of these, pertinence was 100.0. Negatives are the 15 cases in which the correct gene was not ranked # in the genome-phenome analysis (11 in which it was in the gene discovery display (e.g., Fig. 2) and 4 in which it was in the genome-phenome analysis output by not ranked #1)

For the positives, pertinence of the zygosity ranked #1 in the genome-phenome analysis was 100% in 67% of cases (Fig. 3, e.g., the case in Fig. 1). Pertinence was intermediate in 19% of cases and less than 1.45% in 14% of cases.

For the negatives, pertinence of the zygosity ranked #1 in the genome-phenome analysis was less than 1.45% in 93% of cases (1.45% is where the Positives and Negatives curves cross). Pertinence was higher in only one case (the one 90% pertinence value in Fig. 3). In this case, only 4 present findings were used, ones shared very widely among many diseases (microcephaly, motor delay, intellectual disability and autism). Seven absent findings had been used (seizures, deafness, visual impairment, weakens, regression, splenomegaly and high lactate) but the pertinence metric indicated that none of these absent findings had significant influence on the diagnosis, and the top 10 findings recommended by the tool’s usefulness metric were all about facial findings that would have clarified the diagnosis. The ranking of the correct gene would have shifted to #1 if the experimental design had allowed adding pertinent negative facial findings for the *BPTF* monoallelic zygosity, which is the reason that the Gleeson group had settled instead on *MCPH1* biallelic zygosity as the diagnosis for this case.

For the individual interpreting the genome, Fig. 3 gives a good intuitive sense of the meaning of the pertinence value: gene zygosities with 100% pertinence were always pathogenic, other high pertinence scores were typically pathogenic, and non-pathogenic zygosities almost always had very low pertinence.

**Fig. 4:**
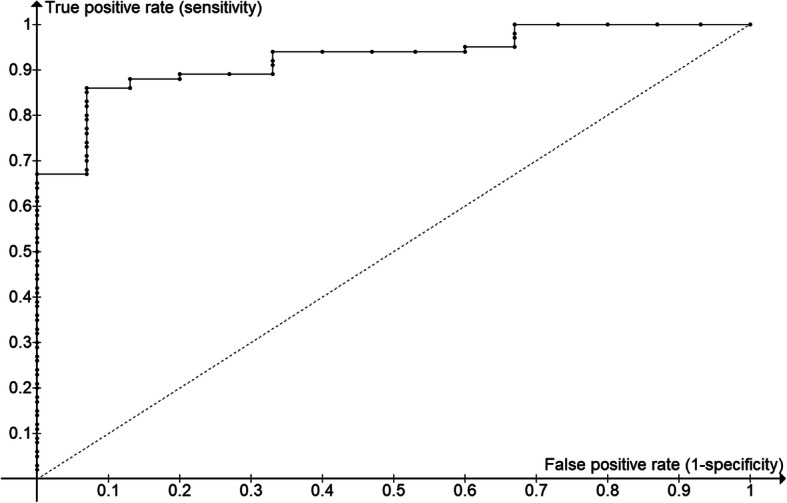
**Receiver operating characteristic (ROC) curve for the diagnostic ability of the analysis**. All 81 cases were ranked by their pertinence scores and the true positive rate and the false positive rate are displayed. The area under the ROC curve is 0.93

More generally, the ability of a metric to indicate a diagnosis is assessed using a Receiver Operating Characteristic (ROC) curve, which assesses the relationship between false positives and false negatives for various values of the metric [20]. The ROC curve for pertinence in these cases is shown in Fig. 4; the area under the ROC curve is 0.93 (perfect, represented by the top left corner, would be 1.00, and random, represented by the dotted line, would be 0.50).

### CNV analysis

When a combined CNV + SNV variant table is analyzed, 3 types of results are shown together, as in Fig. 1: SNV abnormalities (e.g., *NBAS*), single gene abnormalities from CNV areas (e.g., *GLDC* in an 8007 base pair deletion on chromosome 9), and described chromosomal disorders based on the overlap between a CNV detected in the patient and such a described CNV (e.g., Chromosome 16p13.11 deletion). In Fig. 1, the pertinence for each of these is intercompared in a hypothesis-independent way, and here the *GLDC* variants have 100% of the pertinence (green shading of the gene zygosity).

As shown in Table 1, the ability to correctly rank abnormalities in CNV cases was similar to that in SNV cases.

The genome-phenome analysis was designed to also look for associations of large CNVs in patients with the 185 large CNV syndromes curated in the SimulConsult database (chosen as large CNVs with an article in Online Mendelian Inheritance in Man (OMIM), Orphanet Journal of Rare Diseases or GeneReviews). It flagged such associations in many CNV cases (e.g., Chromosome 16p13.11 deletion syndrome in Fig. 1), but no such zygosity findings were ranked #1 in pertinence and no cases with such CNV syndromes being pathogenic were provided by the Gleeson group.

### Gene discovery

Eleven of the 81 cases were “gene discovery” cases: ones in which a gene zygosity for which there was no published human phenotype was determined to be causative (Fig. 2 and Gleeson et al., in preparation). The gene discovery display does not include pertinence because pertinence is defined in terms of a differential diagnosis, and by definition, discovery genes have no association with a human phenotype.

For the 11 gene discovery cases, the number of candidate genes in the gene discovery list having severity equal or greater than the discovery gene chosen by the Gleeson group decreased with the number of individuals sequenced. For SNV cases it decreased from 21.3 for 2 individuals to a perfect 1.0 with 3 or more individuals. The number of candidate genes was higher when genes from CNV regions were included but decreased with the number of individuals (140 for 1 individual, 57 for 3 individuals).

## Discussion

### Systematizing the clinical correlation

The goal of this test of diagnostic decision support was to see if it could convert a long manual process of genome assessment and clinical correlation performed by specialized laboratory personnel to a rapid one that could be performed by a clinician [1–3]. Two additional hypotheses tested here for the first time were whether such an approach can be extended to include CNV analysis and beyond-the-trio cases.

The key result is that in 100% of these 70 cases the correct gene zygosity was identified, and it was ranked #1 in 94% of cases, and #1 - #4 in 100% of cases. Since the computational time is seconds, this provides a tool for clinical correlation of genomic results that can be used by clinicians to arrive at a genomic diagnosis and assess its clinical plausibility with far greater speed and lower cost than a more manual analysis and clinical correlation.

The automated analysis also promotes quality improvement by making it possible to quantitate the value of different components of genomic analysis. This was done here by providing guidance on the interpretation of gene zygosity pertinence scores (Figs. 3, 4) and showing the effect of adding further individuals beyond the trio. This adds evidence-based guidance for determining the optimal number of genomes to order, balancing the costs of testing with those of failure to diagnose.

### CNV analysis as part of genomic analysis

The ability to combine CNV analysis with SNV analysis suggests that the current practice of doing microarray analysis before genomic analysis [4] may result in unnecessary delay and cost, and that the approach used here in which the CNV information is obtained from genomic analysis and the CNV and SNV information are analyzed together could improve speed of diagnosis and reduce costs.

### Hypothesis-independence

A crucial property for such a tool is hypothesis-independence. As in the earlier study [2], the decision support analysis is hypothesis-independent as to the mode of inheritance (e.g., autosomal recessive, compound heterozygote), the number of genes involved, and which clinical findings were most important. Here, when CNV information was added to the variant table and CNV-related abnormalities appeared in the same ranked pertinence list (Fig. 1), the analysis adds a 4th type hypothesis-independence: whether the abnormality is in a CNV or a SNV.

### Use of such a tool in actual clinical practice

Although these results provided a rapid analysis with high accuracy, our analysis of these cases suggests that in actual clinical practice there would be further improvements in performance because of two types of checking done by clinicians:

#### Clinical correlation informed by abnormal gene zygosities

No opportunity was provided in the study design for adding pertinent positives and pertinent negatives after the list of pertinent gene zygosities was available (Fig. 1). This approach was necessary because for many of the cases the clinical descriptions were provided as written records to the Gleeson group, and further information was not easily available. In particular, the number of negative findings listed by the Gleeson group was sparse. However, in actual clinical practice, once the abnormal gene zygosities are available to the clinician who has examined the patient or has access to the full patient record, more information is brought to bear, i.e., useful findings such as those suggested in the software’s useful findings algorithm [16] can be used to add pertinent positive and pertinent negative findings. Decades of studies in medical informatics have shown that an essential element of medical diagnosis is its iterative nature [21, 22] in contrast to a web search that is a one-shot query. The importance of iterative addition of information underscores the relevance of doing clinical correlation after genomic sequencing. As illustrated in Results, adding pertinent negative facial findings to assess the *BPTF* monoallelic zygosity became relevant based on other gene zygosities found. The post-sequencing clinical correlation performed in the Gleeson lab resulted in demotion of an incorrect gene zygosity, but such pertinent negative findings were not provided in the patient clinical data file used in this study. In actual practice, it is always advisable for the clinician to consider further findings. This is most important in the 33% of cases (Fig. 3) in which the pertinence metric was not 100%. In future studies there would be value in modeling such a post-sequencing phase of clinical correlation.

#### Enhancing curation of relevant genes

No opportunity was provided in the study design for adding more information to the database about diseases related to abnormal gene zygosities listed in the genome-phenome analysis. This approach was chosen to avoid biasing the results. However, as discussed in Results, in actual clinical practice, such literature review would be done, particularly in the 33% of cases (Fig. 3) in which the pertinence metric was not 100%. In the 4 cases not ranked #1, doing so raised the pertinence of the gene zygosity identified by the Gleeson group. However, to avoid bias, such changes to the database were not retained in the database or used in this study, even though they met the evidence-based standards for database information changes. In actual clinical practice it is advisable to consider such additional information from the published literature, and those with authority to submit changes to the database can submit such information to the database automatically, thus augmenting the database in a crowd-sourced manner. This is similar to the crowd-sources manner in which ClinVar collects information about variant pathogenicity [23] but submitting SimulConsult database changes takes only seconds using the existing curation interface in the software.

### Reanalysis of genomic results

There is increasing discussion about the importance of reanalyzing genomic results [24]. Three types of new information can impact the clinical correlation:

- **Genotype-phenotype associations**: Hundreds of new genome-phenome correlations and much new information about findings in diseases are discovered each year and added to the database described here in an ongoing curation effort.
- **Patient findings**: The patient’s clinical findings evolve over time, and further laboratory testing is often done.
- **Variant information**: New information about pathogenicity of variant is shared in resources such as ClinVar, and these scores can be used to re-annotate a variant table.

A key advantage of a clinician-focused automated platform is that reanalysis can be done by the clinician in minutes, making use of these 3 forms of new information. Reanalysis of genomic information can now be so routine as to become part of follow-up visits for patients who would otherwise have remained undiagnosed.

In contrast, standard commercial gene panels, often based on a single clinical characteristic such as ataxia, would need to be re-ordered since the panels keep changing. This is prohibitively expensive, and as shown in earlier work [2], it is often not clear before genomic analysis which findings are most important.

### Limitations

The cases analyzed here all had significant neurological findings, reflecting the case mix of the Gleeson group, so the conclusions apply most directly to that group, though it is important to note that most genetic conditions have associated neurologic findings. In this study the range of neurologic findings was not limited to a single manifestation (e.g., seizures), but encompassed a broad range of features, with ~ 10 used per case. This allowed testing of a diverse set of clinical features associated with neurologic findings representative of a typical neurologic or genetic practice setting. A diverse group of genetic disorders with neurologic findings was also included in the analysis. These approaches reduce the risk of introducing bias that would favor the performance of the tool.

The study did not compare to other tools designed for clinical genomic diagnosis such as Phen2Gene [25]. Such comparisons would be important, but because some of the advantages of the tool described here include the ability to use onset age and pertinent negative findings, and the ability to suggest which findings would be useful to include, such studies would need to test multiple clinicians approaching real cases and choosing which findings to include, for which we are developing methodology (Segal, Rahm, Walton and Williams, manuscript in preparation). This study did not compare to tools designed for gene discovery such as VarElect [26], which have capabilities such as knowledge of which gene products interact with other gene products, not included in the tool analyzed here, which was designed primarily for clinical diagnosis.

## Conclusions

Empowering the clinician to do the clinical correlation for genomic analysis is practical using the enhancements described here of a tool already widely used by clinicians for diagnostic decision support. Empowering clinicians in this way restores their central role in genomic analysis. It enables highly effective procedures such as clinical correlation as the final stage of genomic analysis. It makes reanalysis of sequencing so practical as to be routine. The ability to incorporate CNV information in the analysis can save cost and time for testing and analysis.

## Data Availability

The variant data sets supporting the results of this article are available in dbGaP (https://www.ncbi.nlm.nih.gov/gap/) under accession numbers phs000744 and phs001272. Annotated variant table files aggregated for each of the 81 case groups are available on reasonable request from the corresponding author, along with the pedigree and finding files needed to run the analyses. The software runs from https://simulconsult.com, where free trial subscriptions, demo videos and extensive documentation are available.
